# Hydrogen peroxide induced by the fungicide prothioconazole triggers deoxynivalenol (DON) production by *Fusarium graminearum*

**DOI:** 10.1186/1471-2180-10-112

**Published:** 2010-04-15

**Authors:** Kris Audenaert, Elien Callewaert, Monica Höfte, Sarah De Saeger, Geert Haesaert

**Affiliations:** 1 Department Biosciences and Landscape Architecture, Ghent University College/Ghent University Association, Schoonmeersstraat 52, B-9000 Gent, Belgium; 2 Laboratory of Phytopathology, Faculty of Bioscience Engineering, Ghent University, Coupure Links 653, B-9000 Gent, Belgium; 3 Laboratory of Food Analysis, Faculty of Pharmaceutical Sciences, Ghent Univeristy, Harelbekestraat 72, B-9000 Gent, Belgium

## Abstract

**Background:**

*Fusarium *head blight is a very important disease of small grain cereals with *F. graminearum *as one of the most important causal agents. It not only causes reduction in yield and quality but from a human and animal healthcare point of view, it produces mycotoxins such as deoxynivalenol (DON) which can accumulate to toxic levels. Little is known about external triggers influencing DON production.

**Results:**

In the present work, a combined *in vivo/in vitro *approach was used to test the effect of sub lethal fungicide treatments on DON production. Using a dilution series of prothioconazole, azoxystrobin and prothioconazole + fluoxastrobin, we demonstrated that sub lethal doses of prothioconazole coincide with an increase in DON production 48 h after fungicide treatment. In an artificial infection trial using wheat plants, the *in vitro *results of increased DON levels upon sub lethal prothioconazole application were confirmed illustrating the significance of these results from a practical point of view. In addition, further *in vitro *experiments revealed a timely hyperinduction of H_2_O_2 _production as fast as 4 h after amending cultures with prothioconazole. When applying H_2_O_2 _directly to germinating conidia, a similar induction of DON-production by *F. graminearum *was observed. The effect of sub lethal prothioconazole concentrations on DON production completely disappeared when applying catalase together with the fungicide.

**Conclusions:**

These cumulative results suggest that H_2_O_2 _induced by sub lethal doses of the triazole fungicide prothioconazole acts as a trigger of DON biosynthesis. In a broader framework, this work clearly shows that DON production by the plant pathogen *F. graminearum *is the result of the interaction of fungal genomics and external environmental triggers.

## Background

*Fusarium graminearum *is one of the main causal agents of *Fusarium *head blight (FHB) in small grain cereals [1]. Although FHB symptoms have a classical impact on yield, the major concern referred to FHB is the presence of mycotoxins. *Fusarium *spp. are able to produce a plethora of mycotoxins with diverse chemical and biological features [2]. This toxin fingerprint, inherent to the genetics of each individual strain, determines the chemotype of each particular *Fusarium *isolate. *F. graminearum *chemotypes are mainly characterized by type B trichothecenes among which deoxynivalenol (DON), acetyldeoxynivalenol (3-ADON and 15-ADON) and nivalenol (NIV) are the most prevalent [3].

Although the genetic background of type B trichothecene production has been studied elaborately, a coherent view on the production profile of these mycotoxins during infection and colonization of a host is lacking and identifying or understanding mechanisms that regulate the production of these secondary metabolites remains a challenge [4-6]. To date, the role of the type B trichothecene DON during infection and colonization of plants remains a controversial issue. Using DON non-producing *Fusarium *strains, the importance of DON production during spread of the fungus throughout the grain host was demonstrated [4]. In concordance, DON production elicits defence responses in wheat [5]. This role for DON as a virulence factor, actively produced during the infection process, has been confirmed in many other studies [6-8]. Notwithstanding these compelling lines of evidence, other authors uncouple DON production from colonization and aggressiveness [9-11]. The aforementioned controversy illustrates nicely that besides the genotypical derived DON-chemotype, many environmental triggers are crucial to unequivocally delineate the DON-production by a strain of *Fusarium*. The involvement of external influences triggering DON production is further corroborated by research illustrating modulation of DON production by either abiotic factors such as a_w_, temperature, available carbon and/or nitrogen source, and biotic factors such as presence of other fungi [12-16].

The importance of these external triggers in DON production is consolidated by the observation that the production level of mycotoxins in axenic *in vitro *cultures is often orders of magnitude lower than observed during infection and colonization of a host, suggesting that specific host signals are involved in eliciting mycotoxins production. The secondary plant signalling compound hydrogen peroxide (H_2_O_2_), which is involved in plant-fungi interactions, is highlighted as an possible trigger interfering with type B trichothecene production. In previous work with *F. graminearum*, it was demonstrated that exogenously applied H_2_O_2 _at time of spore germination resulted in higher DON and A-DON levels 30 days later [17]. In addition, this DON accumulation was accompanied by an up-regulation of the *tri *gene machinery, responsible for DON biosynthesis [18,19]. Moreover, liquid cultures of *F. graminearum *supplied with H_2_O_2 _started to produce H_2_O_2 _themselves and the kinetics of this paralleled with DON accumulation [19] indicating a link between DON production and oxidative stress. Notwithstanding this clear observation, underlying mechanisms remain elusive. Recently, evidence is brought forward that the response of *Fusarium *to H_2_O_2 _is chemotype dependent. Ponts et al. (2009) observed a reduced NIV production in these chemotypes upon exogenous H_2_O_2 _application while the opposite was observed in DON chemotypes. Furthermore these data suggest that NIV isolates combine this adaptation to oxidative stress with a proliferated virulence [20].

The application of fungicides as possible external triggers for thrichothecene biosynthesis remains a controversial issue. Several authors have described that sublethal concentrations of fungicides trigger thrichothecene biosynthesis [21-23]. Others report opposite results [24,25].

The objective of the present work, was to investigate the influence of three fungicides i.e. prothioconazole (a triazole fungicide), azoxystrobin (a strobilurin fungicide) and prothioconazole + fluoxastrobin, applied at sub lethal concentrations on DON production by *F. graminearum*. Triazoles are known inhibitors of the ergosterol biosynthesis in fungi while strobilurin fungicides inhibit mitochondrial electron transport by binding the Qo site of cytochrome bc1 complex. Where the effectiveness of triazole fungicides against *Fusarium *spp. is a certainty, the activity of strobilurins against *Fusarium *spp. is doubtable.

The hypothesis of a fungicide-induced oxidative stress response as a trigger for DON biosynthesis was evaluated by a combined approach of H_2_O_2 _measurements and application of the H_2_O_2 _scavenger enzyme catalase. Finally, the work was validated on a laboratory scale in an *in vivo *assay using wheat plants. The present work clearly demonstrates the risks of reduced fungicide doses with respect to DON accumulation.

## Results

### Effectiveness of fungicides to inhibit conidial germination and to reduce fungal biomass

**Figure 1:**
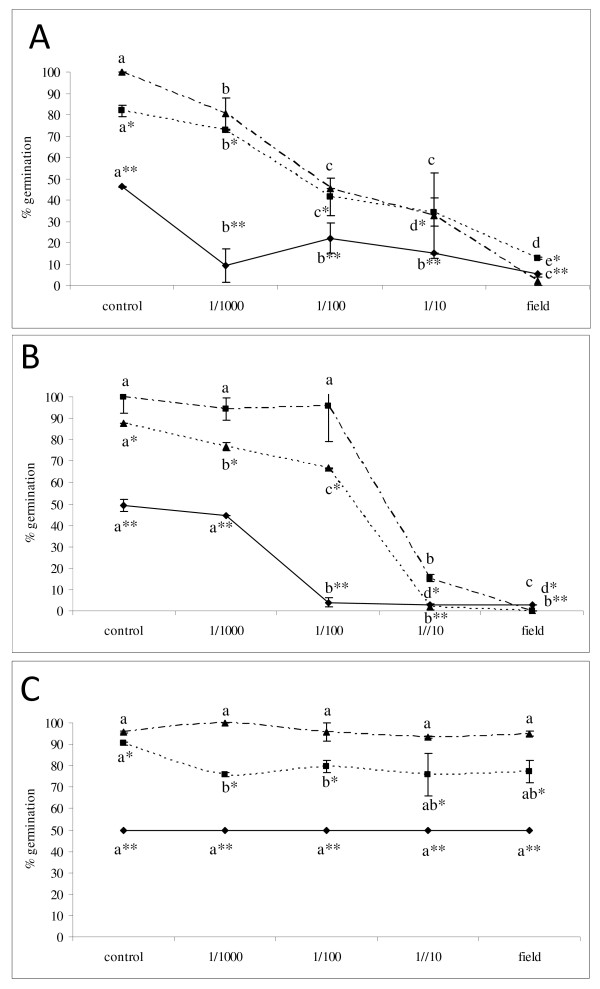
**Effect of prothioconazole + fluoxastrobin (a), prothioconazole (b) and azoxystrobin (c) on conidial germination of *F. graminearum***. Conidia at a concentration of 10^6 ^conidia/ml were challenged with a tenfold dilution series of fluoxastrobin + prothioconazole, azoxystrobin and prothioconazole starting from 0.5 g/l + 0.5 g/l, 0.83 g/l and 0.67 g/l. For each treatment and repetition 50 conidia were scored for their germination and percentage of conidial germination was calculated at 4 h (solid line), 24 h (dashed line) and 48 h (point dashed line) after staining with 0.02% of cotton blue in lactic acid. Experiment consisted of two repetitions for each treatment and the experiment was repeated three times. Graphs represent the average of all three experiments. Different letters at each data point indicate differences from the control treatment at 4 h (**), 24 h (*) and 48 h after analysis with a Kruskall-Wallis and Mann-Whitney test with a sequential Bonferroni correction for multiple comparisons.

Strobilurins and triazoles are among the most frequently used fungicides to respectively control *M. nivale *and *F. graminearum*. Nevertheless, application of these chemicals is often suboptimal due to the short vulnerable period of the pathogen in the field (during anthesis of the host), and environmental factors such as rain and wind. To determine if suboptimal fungicide treatments influence germination of *F. graminearum *conidia and DON production, an *in vitro *assay was set up using a dilution series of azoxystrobin, prothioconazole and fluoxastrobin + prothioconazole. Azoxystrobin did not influence the *F. graminearum *conidial germination at any of the given time points in a concentration-dependent way (Figure 1C). In contrast, prothioconazole effectively inhibited conidial germination at field dose and in dilutions 1/10 and 1/100 but did not have a significant effect at lower doses at time point 48 h (Figure 1B). At time intervals 4 h and 24 h, intermediate concentrations caused a temporary delay in germination. Finally the combination of prothioconazole and fluoxastrobin exhibited fungicidal activity at field concentration and inhibited germination in dilutions 1/100 and 1/100 and displayed no or very little effect in dilution 1/1000 (Figure 1A). Similar results were observed at the level of mycelial radial outgrowth (data not shown).

The effect of the different fungicides on conidial germination was also reflected in the amount of fungal biomass as measured by Q-PCR analysis (Table 1). These Q-PCR data clearly highlighted an effect of prothioconazole and protioconazole + fluoxastrobin on *Fusarium *growth.

**Table 1:** Effect of a tenfold dilution series of prothioconazole, prothioconazole + fluoxastrobin and azoxystrobin on fungal biomass measured by Q-PCR analysis.

	prothio	prothio+catalase*	prothio+fluoxa	prothio+fluoxa+catalase*	azoxy	azoxy+catalase*
control	235.68^a^	194.60^a^	255.68^a^	245.89^a^	251.57^a^	232.45^a^
1/1000	9.42^b^	63.03^b^	76.23^b^	48.17^b^	267.16^a^	230.12^a^
1/100	2.35^c^	31.13^c^	16.58^c^	44.90^b^	250.01^a^	234.93^a^
1/10	2.51^c^	50.02^bc^	LD	LD	254.22^a^	216.00^a^
field	LD	33.47^c^	LD	LD	236.54^a^	170.72^a^

*F. graminearum *biomass expressed as ng/μl. In each run, a no-template control was included. The amount of fungal material was measured based on a standard series of *F. graminearum *DNA ranging from 100 ng/μl down to 3.125 ng/μl which was carried out in triplicate.

Different letters indicate significant differences after analysis with a Kruskall-Wallis Mann-Whitney analysis with P = 0.05

Prothio: prothioconazole; azoxy: azoxystrobin; fluoxa:fluoxastrobin

*: Effect of catalase (1000 U/ml) added at the start of the experiment on the *F. graminearum *biomass.

LD: Lower than detection limit.

### Effect of fungicides on DON production

**Figure 2:**
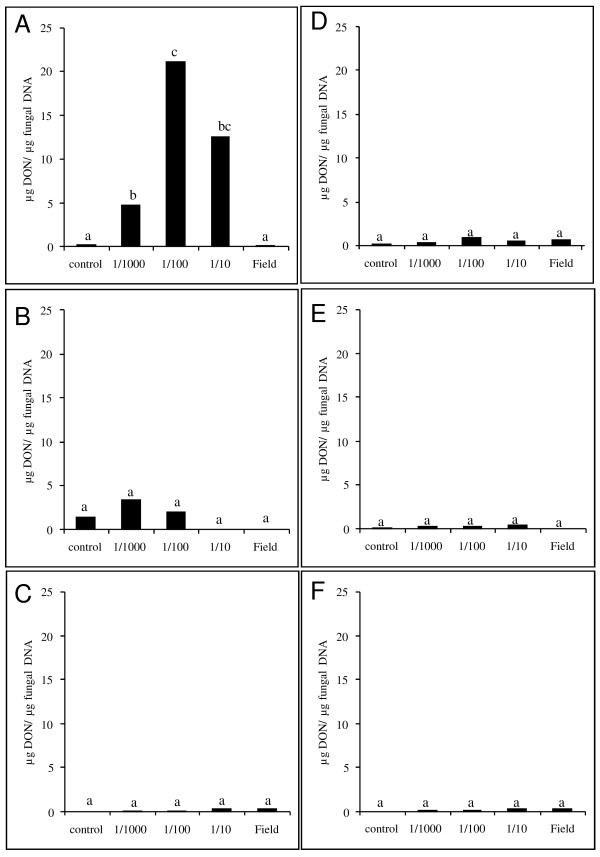
**Effect of prothioconazole + fluoxastrobin (a), prothioconazole (b) and azoxystrobin (c) alone or in combination with catalase (d,e,f) on production of deoxynivalenol (DON) by *F. graminearum***. Conidia at a concentration of 10^6 ^conidia/ml were challenged with a tenfold dilution series of fluoxastrobin + prothioconazole, azoxystrobin and prothioconazole starting from 0.5 g/l + 0.5 g/l, 0.83 g/l and 0.67 g/l in absence (a,b,c) or presence (e,f,g) of 1000 U/ml catalase. DON content in the medium was determined using a competitive ELISA approach 48 h after start of the experiments. Each bar is the result of two pooled samples to reduce variance. The experiment was repeated twice in time of which one representative experiment is shown in the figure. Different letters above bars indicate significant differences after analysis with a Kruskall-Wallis and Mann-Whitney test with a sequential Bonferroni correction for multiple comparisons.

To check whether the effect of the strobilurin fungicides and the triazole fungicide prothioconazole on fungal biomass and germination was paralleled by a reduced production of the type B trichothecene DON, levels of this mycotoxin were measured using a competitive ELISA-approach (Figure 2A, B, C). As expected, application of azoxystrobin did not influence DON production by *F. graminearum *strain 8/1. Remarkably, the combined application of prothioconazole and fluoxastrobin triggered a huge production of DON at the sub lethal doses of dilution 1/10 and 1/100, as early at time point 48 h but not at earlier time points (4 h and 24 h, data not shown). For the sole application of prothioconazole no major effects on DON production were observed since none of the tested concentrations were sub lethal. In an additional experiment using an extra intermediate concentration of 1/50 of the field concentration of prothioconazole, a reduced spore germination of about 50% was observed (data not shown). Concomitant with this observation, this sub lethal dilution resulted in an increased DON production (32 μg/μg of fungal DNA). Hence, application of sub lethal concentrations of respectively prothioconazole + fluoxastrobin and prothioconazole seems to result in the activation of the trichothecene biosynthesis machinery leading to an accumulation of DON as fast as 48 h after the start of the experiment.

### Timely production of H_2_O_2 _precedes DON accumulation in combined strobilurin and triazole fungicide application

**Figure 3:**
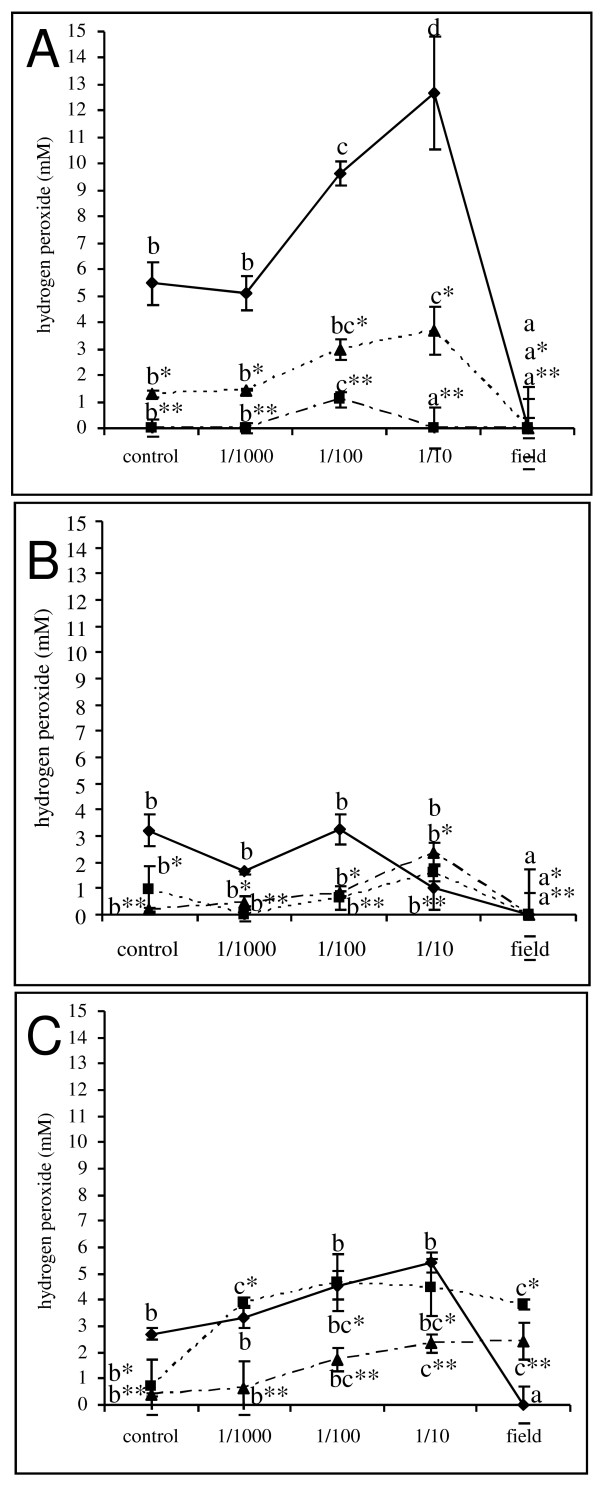
**Effect of prothioconazole + fluoxastrobin (a), prothioconazole (b) and azoxystrobin (c) on extracellular H_2_O_2 _concentrations**. Conidia at a concentration of 10^6 ^conidia/ml were challenged with a tenfold dilution series of fluoxastrobin + prothioconazole, azoxystrobin and prothioconazole starting from 0.5 g/l + 0.5 g/l, 0.83 g/l and 0.67 g/l. H_2_O_2 _was measured at 4 h (solid line), 24 h (dashed line) and 48 h (point dashed line) using TMB (trimethylbenzidine) as a substrate in the presence of an overdose of peroxidase. The H_2_O_2 _concentrations were calculated based on a standard curve included in each experiment. Each data point is the result of three repetitions and the experiments were repeated twice in time. Different letters at each data point indicate differences from the control treatment at 4 h (**), 24 h (*) and 48 h after analysis with a Kruskall-Wallis and Mann-Whitney test with a sequential Bonferroni correction for multiple comparisons.

As several lines of evidence in literature corroborate an important role for reactive oxygen species (ROS) and more specifically H_2_O_2 _in stress responses of fungi, the accumulation of H_2_O_2 _upon fungicide application was monitored in the established *in vitro *germination assay. In these experiments, we unequivocally demonstrated that sole application of respectively azoxystrobin and prothioconazole at the given concentrations did not result in elevated H_2_O_2 _concentrations at any of the time points (Figure 3). In addition, prothioconazole at field dose resulted in lower H_2_O_2 _concentrations than those observed in control samples possibly reflecting the reduction in microbial metabolic activity due to the application of the fungicide. Sub lethal dilutions of the combined application of fluoxastrobin + prothioconazole (i.e. 1/10 and 1/100) resulted in an increased H_2_O_2 _content in the medium compared to the control and the other treatments as fast as 4 h after the start of the germination assay. Although the increase at concentration 1/100 was less proliferate than the increase at concentration 1/10 of the field dose of fluoxastrobin + prothioconazole, it was consistent in all performed experiments. Moreover, this peak in H_2_O_2 _disappeared or was less proliferated at later time points 24 h and 48 h. These findings strongly suggest that timely production of H_2_O_2 _triggers the trichothecene biosynthesis machinery to produce DON in sub lethal fungicide treatments.

**Figure 4:**
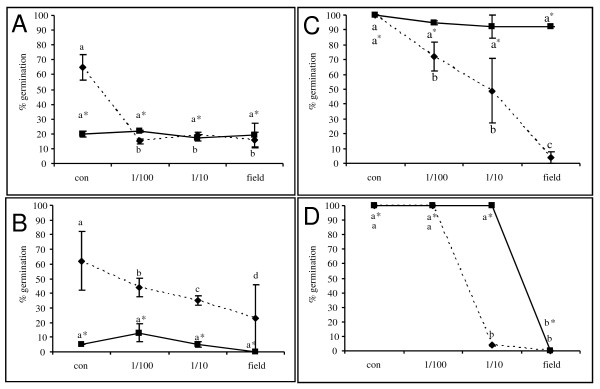
**Effect of prothioconazole + fluoxastrobin (a, c) and prothioconazole (b, d) in absence (dashed line) or presence (solid line) of exogenous catalase on the germination of *F. graminearum *conidia after 4 h (a, b) and 48 h (c,d)**. Conidia at a concentration of 10e^6 ^were challenged with a tenfold dilution series of fluoxastrobin + prothioconazole, azoxystrobin and prothioconazole starting from 0.5 g/l + 0.5 g/l, 0.83 g and 0.67 g/l. At the beginning of the experiment, catalase (1000 U/ml) was added to the germinating conidia. For each treatment and repetition 50 conidia were scored for their germination after staining with 0.02% of cotton blue in lactic acid and percentage of conidial germination was calculated. This experiment was repeated twice in time. Different letters at each data point indicate differences from the control treatment after analysis with a Kruskall-Wallis and Mann-Whitney test with a sequential Bonferroni correction for multiple comparisons.

**Figure 5:**
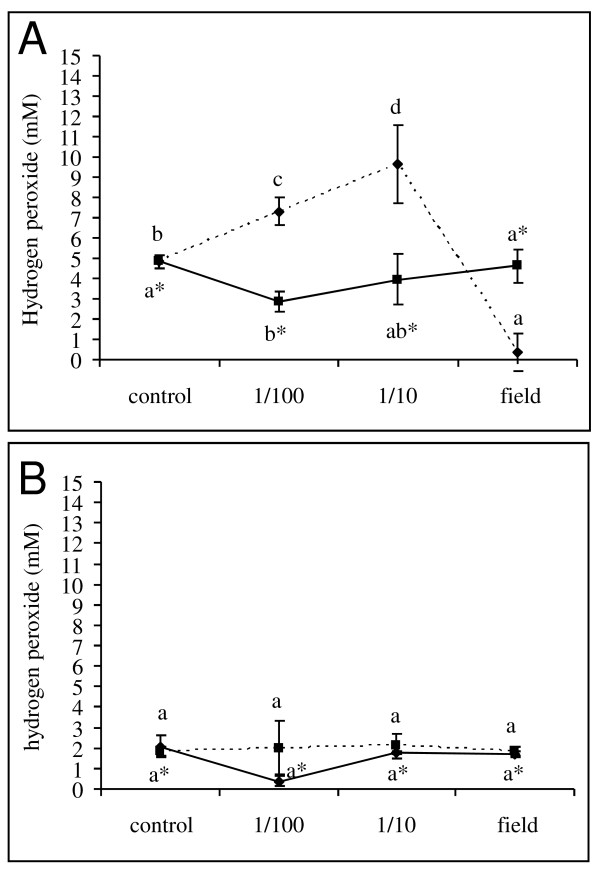
**Effect of a combined application of catalase and respectively prothioconazole + fluoxastrobin (a) and prothioconazole (b) on extracellular H_2_O_2 _concentrations at 4 h after fungicide application**. Conidia at a concentration of 10^6 ^conidia/ml were challenged with a tenfold dilution series of fluoxastrobin + prothioconazole, azoxystrobin and prothioconazole starting from 0.5 g/l + 0.5 g/l, 0.83 g and 0.67 g/l in the absence (dashed line) or presence of 1000 U/ml catalase (solid line). H_2_O_2 _was measured at 4 h using TMB (trimethylbenzidine) as a substrate in the presence of an overdose of peroxidase. The H_2_O_2 _concentrations were calculated based on a standard curve included in each experiment. Each data point is the result of three repetitions and the experiments were repeated twice in time. Different letters at each data point indicate differences from the control treatment after analysis with a Kruskall-Wallis and Mann-Whitney test with a sequential Bonferroni correction for multiple comparisons.

To further examine the role of H_2_O_2 _in fungicide-induced stress, exogenous catalase was added together with the fungicidal treatment. At 4 h after application, catalase resulted in a reduced germination rate (Figure 4A, B) compared to all non-catalase treatments. In addition, at later time points, the application of catalase partially abolished the fungicidal effect of prothioconazole + fluoxastrobin (Figure 4C) and of prothioconazole (Figure 4D) at both the level of conidial germination and fungal biomass (Table 1). No effect was observed in the treatment with azoxystrobin (data not shown). In addition, this partial loss of fungicidal effect due to the application of catalase was accompanied by the disappearance of the H_2_O_2 _peak previously observed in the prothioconazole + fluoxastrobin treated samples at 4 h after application of prothioconazole (Figure 5A). No peak was observed in the treatment with sole application of prothioconazole (Figure 5B). At later time points, no H_2_O_2 _accumulation was observed in none of the treatments (data not shown). Finally, completely in line with these observations, the disappearance of the H_2_O_2 _trigger at 4 h due to the application of catalase resulted in DON production comparable to control treatments (Figure 2D, E, F).

### Stress-induced H_2_O_2 _accumulation upon fungicide application is necessary and sufficient as a trigger to induce DON

**Figure 6:**
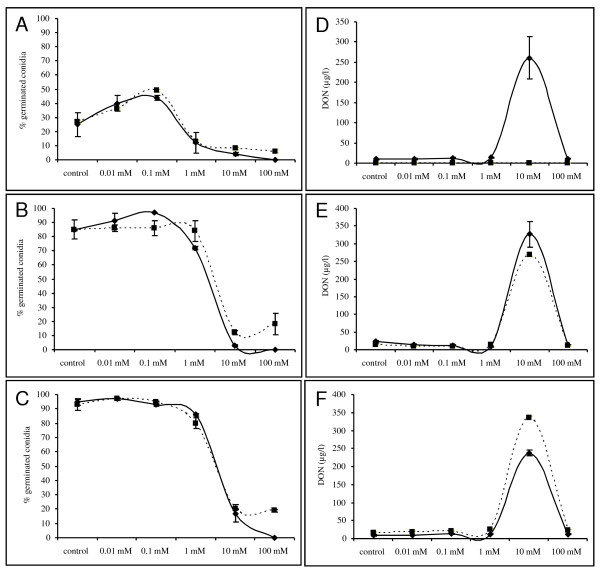
**Effect of exogenously applied H_2_O_2 _on germination (a, b, c) of *F. graminearum *and DON production (d,e,f) after 4 h (a and d), 24 h (b and e) and 48 h (c and f)**. Conidia at a concentration of 10^6 ^conidia/ml were challenged with a tenfold dilution series of H_2_O_2_. For each treatment and repetition 50 conidia were scored for their germination after staining with 0.02% of cotton blue in lactic acid and percentage of conidial germination was calculated. DON content in the medium was determined using a competitive ELISA approach. Each treatment was measured in duplicate and the experiment was repeated twice in time (dashed and solid line represent the two experiments).

To further decipher a direct link between H_2_O_2 _at one hand and the production of the mycotoxin DON at the other hand, the accumulation of DON was monitored upon exogenously single pulse application of H_2_O_2_ranging from 0.01 mM up to 100 mM. H_2_O_2 _influenced germination of *F. graminearum *conidia in a concentration-dependent manner (Figure 6). As early as 4 h after the start of the assay, exogenously application of H_2_O_2 _at concentrations from 1 mM up to 100 mM retarded or stopped conidial germination. The sub lethal concentration of 10 mM H_2_O_2 _induced DON production as fast as 4 h after application of H_2_O_2 _in one of the experiments. In the other experiment, 4 h was probably just too early to observe the increased DON production and in this experiment, the increment in DON was observed at 24 h. The ability of 10 mM H_2_O_2 _to initiate DON production is in concordance with H_2_O_2 _concentrations induced by sub lethal prothioconazole concentrations (Figure 3A). At later time points, DON did not further accumulate and concentration remained the same for the subsequent 24 and 48 h time points. This effect of H_2_O_2 _on DON production was confirmed by an experiment in which H_2_O_2 _was eliminated from the well plates by exogenously applied catalase. This resulted in a fall-back of the DON production in the 10 mM H_2_O_2 _treatment to levels comparable to control wells (data not shown). Finally, surprisingly, low concentrations of H_2_O_2 _facilitated conidial germination compared to control samples. Indicating the necessity of low levels of H_2_O_2 _in optimal germination of conidia and proliferation of fungal cells.

### Sublethal prothioconazole + fluoxastrobin application triggers DON production *in vivo*

**Figure 7:**
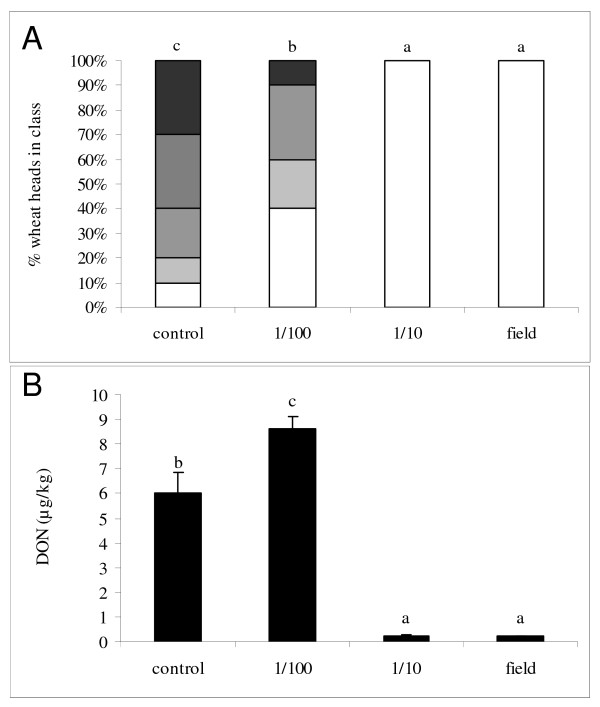
***In vivo *effect of prothioconazole + fluoxastrobin on symptoms of *F. graminearum *(a) and DON content (b) after point inoculation of wheat ears 14 days after infection**. Wheat ears (variety Cadenza) were inoculated with two droplets of 20 μl of conidia at a concentration of 10e^6 ^conidia/ml. Infection spots were indicated with a marker. Ears were subsequently treated with a tenfold dilution series of fluoxastrobin + prothioconazole starting from 0.5 g/l + 0.5 g/l. For each treatment, 10 plants were assessed for *Fusarium *symptoms. This experiment was repeated twice in time with analogous results. The figure represents one representative experiment. Different letters at each data point indicate differences from the control treatment after analysis with a Kruskall-Wallis and Mann-Whitney test with a sequential Bonferroni correction for multiple comparisons.

In an *in vivo *case study with azoxystrobin and prothioconazole + fluoxastrobin, the effect of sub lethal fungicide concentrations on growth and DON production was verified on wheat plants (variety Cadenza) during anthesis. A point inoculation with *F. graminearum *clearly led to typical *Fusarium *symptoms 14 days after inoculation (Figure 7). In the treatment with azoxystrobin, no reduction of symptoms was observed (data not shown) which is in concordance with the previously described *in vitro *data. Application of prothioconazole + fluoxastrobin resulted in a complete control of *Fusarium *at field dose or dilution 1/10 (Figure 7A). At concentration 1/100 symptoms were apparent although they were less proliferate than in the inoculated control plants pointing to a sub lethal concentration. Parallel with the symptom evaluation, DON content was determined in the wheat ears. No DON was apparent in treatments with field dose or dilution 1/10. However, a significant increase in DON content was observed in ears originating from the 1/100 treatment compared to the control treatment (Figure 7B) which is in concordance with the *in vitro *observations.

## Discussion

In an effort to broaden our understanding of external triggers influencing the DON production machinery of *F. graminearum*, the effect of strobilurin and triazole fungicides on DON production was investigated. Our results demonstrate that prothioconazole, a triazole fungicide, has good control capacities culminating in reduced vegetative radial outgrowth, a reduced conidial germination and a reduction of *F. graminearum *biomass. Triazoles are known inhibitors of the ergosterol biosynthesis in fungi and have been described for their good control capacities against *Fusarium *spp [21].

On the contrary, the strobilurin fungicide azoxystrobin was not able to induce a reduction in radial outgrowth, spore germination and fungal biomass. Strobilurin fungicides inhibit mitochondrial electron transport by binding the Qo site of cytochrome bc1 complex. Although the effectiveness of strobilurins against *Fusarium *spp. is doubtable, they have been reported to be effective against *F. culmorum *[24] Apparently, *F. graminearum *is very resistant to this type of fungicides. Resistance to strobilurin fungicides has been reported in many species to be associated with a single amino acid replacement at position 143 of the cytochrome b gene [26-28]. Although this mechanism was recently described in *Microdochium nivale *it has not yet been described in *F. graminearum*. We assume that the observed resistance is therefore possibly a consequence of the activation of a respiratory chain using an alternative oxidase (AOX) bypassing complexes III and IV in the cytochrome mediated pathway. Activity of this AOX mediates electron transfer directly from ubiquinol to oxygen. Kaneko and Ishii (2009) demonstrated that *F. graminearum *acts very rapidly upon strobilurin application by the activation of AOX whereas *M. nivale*, a fungal species susceptible to strobilurins, reacted slowly with a retarded moderate activation of this enzyme [29].

Since the generation of reactive oxygen species such as H_2_O_2 _is a hallmark of an oxidative stress response, extracellular H_2_O_2 _was measured upon fungicide application in an *in vitro *assay. Unexpectedly, application of strobilurin fungicides did not result in an increased extracellular H_2_O_2 _formation, which is at first sight, contradictory to previous findings by Kaneko and Ishii (2009) who found an increased production of H_2_O_2 _upon strobilurin application. However it is important to notice that in the present work the H_2_O_2 _released in the medium was measured whereas Kaneko and Ishii (2009) focused on intracellular H_2_O_2_. Remarkably, the application of sub lethal doses of prothioconazole or the combination of prothioconazole amended with fluoxastrobin resulted in a boosted H_2_O_2 _production as fast as 4 h after application. This prompt production disappeared at later time points. In addition, a clear induction of DON production was observed 48 h after application of sub lethal prothioconazole + fluoxastrobin concentrations. This induction of DON was confirmed in an *in vivo *experiment in which flowering wheat plants were infected with *F. graminearum *and subjected to a sub lethal dose of prothioconazole + fluoxastrobin. Previous work on *F. culmorum *demonstrated no or a negative effect of several strobilurins and triazoles on DON production [24] so the observed phenomenon of an increased DON production by *F. graminearum *induced by sub lethal concentrations of triazole fungicides might be a strain- or species-specific phenomenon.

It is tempting to speculate whether this accumulation of DON is the consequence of the preceding accumulation of H_2_O_2 _as such being the first link in a signalling cascade activated upon sub lethal triazole treatment. Although this key role of H_2_O_2 _is not unambiguously demonstrated in the present study, the amount of evidence is compelling: H_2_O_2 _precedes accumulation of DON, combined application of catalase (eliminating H_2_O_2 _from the medium) inhibited DON accumulation. In addition, the application led to a reduced activity of the triazole fungicide. Application of H_2_O_2 _to *F. graminearum *cultures led to a reduced germination and prompt induction of DON biosynthesis 4 h after H_2_O_2 _application. This additional experiment proves that H_2_O_2 _accumulation is necessary and sufficient to initiate DON production. The activation of the DON biosynthesis machinery by H_2_O_2 _is in concordance with previous observations by the group of Barreau [17,19,20] who demonstrated that exogenously applied H_2_O_2 _by repeated single or pulse-feeding resulted in accumulation of DON. However, these authors only monitored increases in DON at late time points such as 10 to 30 days after H_2_O_2 _application whereas we observe a clear prompt activation of DON production within hours. From a physiological point of view the effect of H_2_O_2 _during the initial germination events is logic and in line with the physiology of an in field *F. graminearum *infection: H_2_O_2 _is one of the key regulators in the plant defense system upon pathogen attack [30]. Therefore, this molecule is encountered frequently and at early time points by the pathogen in the interaction with its host. Previous work by the group of John Manners demonstrated beautifully that DON itself can induce hypersensitive cell death and H_2_O_2 _during infection [5] and as such underpinning the interaction between both molecules.

Astonishingly, very low concentrations of H_2_O_2 _promoted conidia germination rate where a reduction was expected. We hypothesize that during germination events, very small amounts of H_2_O_2 _are beneficial and necessary in the primordial germination- and hyphal extension events. It is known that H_2_O_2 _is necessary in *de novo *synthesis of cell wall and membrane components during germination and hyphal extension. Indirect evidence for this was provided by Seong et al (2008) who observed high activities of the peroxisomes at the onset of spore germination [31] The need for basal H_2_O_2 _is subscribed by the observation that catalase treatment results in a reduced spore germination at very early time points in germination. In several independent studies, it was demonstrated that reactive oxygen species such as H_2_O_2 _are key players and crucial in the regulation of cell differentiation in microbial eukaryotes [32,33]. In accordance with this, it was demonstrated that NADPH oxidases which generate reactive oxygen are decisive in fungal cell differentiation and growth in a model system using *Neurospora crassa *[34].

Taken together, these results not only reinforce the hypothesis that H_2_O_2 _can induce DON biosynthesis but also suggest that DON accumulation induced by sub lethal triazole application is mediated through an increased production or release of H_2_O_2 _into the medium rendering a physiological interface of H_2_O_2 _influencing DON production. It is tempting to speculate on the mechanistics behind these observations. We hypothesize that due to the inhibition of ergosterol biosynthesis by the application of triazole fungicides, an increased cell permeability results in the increased release of H_2_O_2 _in the medium which in turns activates the trichothecene biosynthesis machinery. Indeed, although H_2_O_2 _is a very reactive molecule which can diffuse freely across bio membranes, it has been shown in a *Sacharomyces *model system that organisms prevent H_2_O_2 _diffusion [35,36]. This hypothesis is subscribed by accumulating indirect evidence in many other fungi. As such in *Candida *ergosterol depletion increases vulnerability to phagocytic oxidative damage [37]. In *Sacharomyces *it was demonstrated using ergosterol knock out mutants that ergosterol depletion results in a changed biophysical property of the plasma membrane leading to an increased permeability towards H_2_O_2_[38].

Although beyond the scope of the present paper it is important to notice that triazole fungicides on their own can generate H_2_O_2 _*in planta *as an intermediate metabolite in plants through activation of antioxidant systems [39] generating as such a greening effect which results in a retardation of the senescence [40]. The effect of this physiological induced H_2_O_2 _*in planta *on DON production by an invading *F. graminearum *is till now not studied and certainly needs more attention in the future.

## Conclusions

In the present work it was shown that sub lethal prothioconazole concentrations resulted in a significant increase in DON production by *F. graminearum *in a combined approach of an *in vitro *assay and an artificial infection trial. In the *in vitro *assay, the stimulated DON production was preceded by a prompt induction of H_2_O_2 _suggesting that the proliferated DON production was induced by an oxidative stress response in the fungus. This hypothesis was confirmed by exogenous application of catalase which abrogated the elevated DON production observed at the sub lethal doses of prothioconazole. In a broader framework, this work clearly shows that DON production by the plant pathogen *F. graminearum *is the result of the interaction of fungal genomics and external triggers. Further work is needed to characterise the effect of these external triggers influencing DON biosynthesis. This work will certainly lead to a better insight into factors that influence DON production under field conditions.

## Methods

### Fungal Material, induction of conidia, conidia suspension and conidia counting

A GFP transformant of *Fusarium graminearum *strain 8/1 [41] was grown on potato dextrose agar (PDA) for 7 days at 20°C and kept at 4°C upon use in the germination assays. Conidia of *F. graminearum *were obtained by incubating a mycelium plug on a PDA plate for 7 days under a light regime of UV/darkness (12 h 365 nm 10 W/12 h). Macroconidia were harvested by adding distilled water amended with 0.01% of Tween20 to the fully grown PDA plates and by rubbing the conidia-bearing mycelium with a spatula. Conidia were counted and diluted to a final concentration of 10e6 conidia/ml. In the germination assays, fungal conidia were visualised using a 0.02% cotton blue solution prepared in lactic acid.

### *In vitro *growth and germination assay, exogenous application of fungicides and H_2_O_2_

In the present study, 3 fungicides were used i.e. fluoxastrobin+prothioconazole, azoxystrobin and prothioconazole. Field doses of each fungicide were the point of departure for the *in vitro *assay. The field dose of each fungicide differed according to the manufacturers instructions and mounted to 0.5 g/l + 0.5 g/l, 0.83 g and 0.67 g for respectively fluoxastrobin+prothioconazole, azoxystrobin and prothioconazole.

In experiments aiming to measure fungal biomass and conidia germination, a ten-fold dilution series of these three fungicides was prepared to obtain a final concentration of 1/1000, 1/100, 1/10 and field dose of each fungicide in the 24-well plates in which the assay was executed. In these wells, 250 μl of conidial suspension was added and amended with 250 μl of the fungicide dilution. These wells were incubated at 20°C. Each treatment consisted out of 2 repetitions and the experiment was repeated three times independently in time. Control treatments consisted of 250 μl of spore suspension and 250 μl of distilled water.

H_2_O_2 _was applied once at the beginning of the germination trials in a final concentration ranging from 0.01 mM, 0.1 mM, 1 mM up to 10 mM. 250 μl of H_2_O_2 _solution was added to 250 μl of spore suspension. Each treatment consisted out of 2 repetitions and the experiment was repeated three times. Control treatments consisted of 250 μl of spore suspension and 250 μl of distilled water.

### Infection of wheat plants and application of fungicides *in vivo*

*F. graminearum *macroconidia were obtained and harvested as previously described. A conidia suspension of 10e6 conidia/ml was prepared. A dilution series of fluoxastrobin and azoxystrobin + prothioconazole was prepared starting from the field dose as mentioned in the *in vitro *assays. Ten ears of wheat plants at flowering stage (Zadok's stage 60) were infected with 2 droplets of 20 μl of conidia suspension. Subsequently, the infected wheat plants were sprayed with fungicide dilutions till run off and placed in a growth chamber at 22°C under a relative humidity of 100% for 2 days to guarantee the conidial germination and penetration. After 2 days, the plants were incubated for 12 days in a growth chamber at 22°C under a light regime of 16 h light/8 h dark. Fourteen days after inoculation, the infection was assessed based on the surface of the ear covered with *Fusarium *symptoms:1 = healthy; 2 = up to 25%; 3 = 25 to 50%; 4 = 50 to 75%; 5 = 75 to 100% of the ear covered with symptoms. The experiment was repeated twice in time.

### DNA extraction and fungal quantification using a Q-PCR approach

To quantify the amount of *Fusarium *biomass in the *in vitro *assays, fungal biomass retrieved from each individual well was centrifuged and supernatant was eliminated. The pellet freeze-dried for 6 h at -10°C and 4 h at -50°C (Christ Alpha 1-2 LD Plus, Osterode, Deutschland). Samples were stored at -20°C upon extraction.

DNA extraction was performed as previously described by Audenaert *et al*. (2009) [42] based on the method established by Shaghai and Mahroof et al. (1989) [43]. For PCR, amplification of the EF1α gene, the forward primer FgramB379 (5'-CCATTCCCTGGGCGCT-3') and the reverse primer FgramB411 (5'-CCTATTGACAGGTGGTTAGTGACTGG-3') were used [44]. The real-time PCR mix consisted of 12.5 μl 2 × SYBR Green PCR Master Mix (Stratagene), 250 nM of each primer, 0.5 μg/μl bovine serum albumin (BSA) and 2 μl of template DNA. PCR was performed on a 7000 series Detection System (Applied Biosystems) using the following PCR protocol: 2 min at 50°C, 10 min at 95°C, 40 cycles of 95°C for 15 s and 62°C for 1 min followed by a dissociation analysis at 55°C to 95°C.

A standard curve was established in threefold using a twofold dilution series of pure fungal DNA from 100 ng up to 3.125 ng. The amount of fungal DNA was calculated from the cycle threshold (Ct) and the amount of fungal material in control samples.

### Measurement of H_2_O_2 _and DON, application of catalase

H_2_O_2 _formation in the fungicide experiments was measured 4 h, 24 h and 48 h post inoculation using a TMB (trimethylbenzidin) assay. This assay is based on the conversion of TMB to a blue stain upon reaction with H_2_O_2 _in the presence of peroxidases. 250 μl of the conidia suspension was removed from a well and amended with an excess of 100 μl horse radish peroxidase (500 U/ml) and 150 μl of TMB (1 mg/ml). TMB was dissolved in 100% ethanol and the stock solution of 1 mg/ml was prepared in 50 mM of Tris-acetate buffer (pH 5.0). H_2_O_2 _formation was determined by measuring the absorbance at 620 nm in duplicate in each time point and in two independent experiments. In each experiment, a standard curve of pure H_2_O_2 _was added in a concentration range of 0.01 mM up to 100 mM. The H_2_O_2 _formed in the *in vitro *assay was calculated based on this standard curve.

DON concentration was measured by ELISA using the Veratox DON 5/5 kit (Biognost, Neogen, Leest, Belgium). The lower limit of detection was 0.1 ppm. A standard curve was established using 0, 0.25, 0.4, 1 and 2 ppm DON. The ELISA kit provides 100% specificity for DON. 200 μl of the conidia suspension was removed from each well. Two repetitions per treatment were pooled and subsequently centrifuged to eliminate the fungal pellet. 100 μl of this supernatant was used for further analysis in the ELISA assay. Experiments in which DON content was measured were repeated twice in time with two repetions per experiment and treatment. In the *in vivo *experiments, 1 g of grains was ground and extracted in 10 ml of distilled water. Subsequently, the extract was analyzed by ELISA as described above. The DON content was measured in five fold.

In the *in vitro *experiments using catalase, 125 μl of Catalase from bovine liver (Sigma, Bornem, Belgium) was added to the wells to a final concentration of 1000 U/ml. In the experiments where catalase was applied, 250 μl of conidia were amended with 125 μl of fungicides. Care was taken that the final concentration of the fungicides was the same as aforementioned in the other studies.

### Data analysis

Differences in DON levels, H_2_O_2 _content, disease assessment, germination and fungal diameter were detected using a non-parametric Kruskall-Wallis and Mann-Whitney test with a sequential Bonferroni correction for multiple comparisons. Differences between DON levels and disease severity were considered at P = 0.05/(n-1) with n the number of cases in the study. All data were analyzed using SPSS-software (Originally: Statistical Package for Social Sciences) version 15.0 for WindowsXP.

## Authors' contributions

KA conceived of the study, carried out most of the *in vitro *assays and drafted the manuscript. EC carried out the immunoassays and helped with the *in vitro *assays partim conidial germination. GH, MH and SDS coordinated and helped to draft the manuscript. All authors read and approved the final manuscript.
